# Metformin regulates lipid metabolism in a canine model of atrial fibrillation through AMPK/PPAR-α/VLCAD pathway

**DOI:** 10.1186/s12944-019-1059-7

**Published:** 2019-05-10

**Authors:** Fan Bai, Yaozhong Liu, Tao Tu, Biao Li, Yichao Xiao, Yingxu Ma, Fen Qin, Jing Xie, Shenghua Zhou, Qiming Liu

**Affiliations:** Department of Cardiology/Cardiac Catheterization Lab, The Second Xiangya Hospital, Central South University, No.139 Middle Renmin Road, Furong District, Changsha, 410011 Hunan China

**Keywords:** Atrial fibrillation, Lipid metabolism, AMPK, Metformin

## Abstract

**Background:**

Atrial lipid metabolic remodeling is critical for the process of atrial fibrillation (AF). Abnormal Fatty acid (FA) metabolism in cardiomyocytes is involved in the pathogenesis of AF. MET (Metformin), an AMPK (AMP-activated protein kinase) activator, has been found to be associated with a decreased risk of AF in patients with type 2 diabetes. However, the specific mechanism remains unknown.

**Methods:**

Fifteen mongrel dogs were divided into three groups: SR, ARP (pacing with 800 beats/min for 6 h), ARP plus MET (treated with MET (100 mg/kg/day) for two weeks before pacing). We assessed metabolic factors, speed limiting enzymes circulating biochemical metabolites (substrates and products), atrial electrophysiology and accumulation of lipid droplets.

**Results:**

The expression of AMPK increased in the ARP group and significantly increased in the MET+ARP group comparing to the SR group. In the ARP group, the expressions of PPARα、PGC-1α and VLCAD were down-regulated, while the concentration of free fatty acid and triglyceride and the lipid deposition in LAA (left atrial appendage) increased. Moreover, AERP and AERPd have also been found abnormally in this process. Pretreatment with MET before receiving ARP reversed the alterations aforementioned.

**Conclusions:**

The FA metabolism in LAA is altered in the ARP group, mainly characterized by the abnormal expression of the rate-limiting enzyme. Metformin reduces lipid accumulation and promotes β-oxidation of FA in AF models partially through AMPK/PPAR-α/VLCAD pathway. Our study indicates that MET may inhibit the FA lipid metabolic remodeling in AF.

## Background

Atrial fibrillation (AF), the most common sustained arrhythmia, is well known to be associated with significant morbidity and mortality. The initiation and progression of AF are caused by atrial remodeling, including structural, electrical and contractile remodeling. All of these have been shown to contribute continuously to the self-perpetuating nature of AF (‘AF begets AF’) [1, 2]. Emerging evidence suggests that metabolic impairment is important for AF pathophysiology [3, 4]. Fatty acids (FA) are the main substrate for ATP production in cardiomyocytes, and mitochondrial β-oxidation of long-chain fatty acids is the main pathway for energy-producing. Very-long chain acyl-CoA dehydrogenase (VLCAD) is one of the most important acyl-CoA dehydrogenases with various chain length specificities and catalyzes the initial step of mitochondrial fatty acid oxidation (FAO) in the human heart. Our previous study showed that VLCAD [5] were downregulated in human persistent AF.

Peroxisome proliferator-activated receptor-α (PPAR-α), a member of the nuclear hormone receptor superfamily, is a critical regulator of myocardial energy metabolism. PPAR-α is preferentially expressed in tissues with high FA utilization like heart and muscle [6]. Energy utilization in the heart is transcriptionally controlled in part by the PPAR family and their coreceptors/coactivators [7]. Mechanistically, PPAR-훼 heterodimerize with the RXR-훼 and coactivators (e.g., PGC-1훼) and repressors (e.g., nuclear receptor corepressor, NCoR) to regulate the transcription of genes involved in energy regulation and lipid metabolism [8–10].

AMP-activated protein kinase (AMPK), a sensor of metabolic stress, regulates various crucial physiological and pathological cellular events. AMPK-mediated phosphorylation of PGC-1α stimulates mitochondrial biogenesis and improves fatty acids metabolism [11]. Accumulating evidence show that alterations of AMPK are involved in the pathogenesis of AF [12–14]. Metformin, a common antidiabetic drug, has been repeatedly shown to possess a cardioprotective role in permanent myocardial infarction (MI) [15], ischemia/reperfusion (I/R) injury [16] and hypertrophy [17], not only via its antidiabetic effects but also through the activation of AMPK.

In patients with type 2 diabetes [18], metformin is associated with a decreased risk of AF, but the mechanism remains unclear. Studies have shown that AMPK regulates PPARα and PGC-1α activity [19, 20], and regulate mitochondrial biogenesis and energy metabolism. Thus, we hypothesize that metformin improves atrial lipid metabolic remodeling through the AMPK/PPAR-α/VLCAD signaling pathway and decrease the incidence of AF. A canine model of acute AF induced by ARP, as described in a previous study [21], was created to investigate our hypothesis.

## Materials and methods

### Group setting

Fifteen adult male mongrel dogs weighing 15-20 kg were bred and supplied by the Experimental Animal Center of Medical College of Wuhan University. All of the dogs were randomly assigned to three groups as follows: (i) SR group (*n* = 5), (ii) atrial rapid pacing (ARP) group (n = 5), and (iii) ARP plus MET group (n = 5). Dogs in SR group and ARP group were treated with a normal diet for two weeks. Dogs in ARP plus MET group were treated with normal diet and oral metformin at 100 mg/kg/day for two weeks as described previously [22]. Dogs in ARP and ARP plus MET group received right atrial rapid pacing for 6 h, while dogs in SR group attain the same procedure but without pacing.

### Canine model of acute atrial fibrillation

All dogs were intravenously anesthetized with sodium pentobarbital (30 mg/kg) and ventilated with an adjustable ventilator (MAO01746, Harvard Apparatus, Holliston, USA). Intravenous injections of additional sodium pentobarbital (2 mg/kg) were conducted at the end of each hour during the procedure. The left femoral vein was cannulated to allow infusion of 0.9% saline (50–100 mL/h) for replacement of spontaneous fluid losses. Standard electrocardiogram (ECG) leads were continuously recorded. All the Data were recorded on a computerized electrophysiology system (Lead 2000B; Jinjiang Inc., Sichuan, China).

The acute AF model was induced by RAP, as described in a previous study [21]. A programmable stimulator (Grass-S88, Astro-Med, West Warwick, RI) was used to deliver RAP to the right atrial appendage (1200 beats per minute, 2 diastolic thresholds) to stimulate AF. Continuous stimulation (20 Hz, 0.1 millisecond square waves) was applied for 6-h RAP to each dog in the ARP and ARP + MET groups. Dogs in SR group process the same procedure but without pacing. Parts of tissue samples taken from the LAA were quickly frozen in liquid nitrogen which were later used for chemical analysis; The left samples were immersed in 4% polyformaldehyde for 24 h and then embedded in paraffin for histological analyses.

### Lipid content in LAA tissues

The free fatty acid (FFA) and triglyceride content were measured by using the concentration measurement kits obtained from Jiancheng Biological Technical Institute (China). All procedure followed biochemical kit instructions. The accumulation of lipid droplets in cardiac myocytes was detected by the Oil Red O staining kit (AS1083, ASPEN). Briefly, 10 μm frozen sections were obtained from a Leica CM1850, Germany and dyed with the Oil red O reagent at room temperature for 5–10 min. And then, we dyed the sections with hematoxylin. The lipid droplets are shown in red and the nuclei in blue. Images were obtained from Zeiss Imager DI microscope with a Zeiss AxioCam MRc5 color camera (Carl Zeiss, Oberkochen, Germany). and the relative density was tested by Image-Pro plus 6.

### Western blot analyses

Canine LAA tissues were lysed with RIPA Lysis Buffer (ASPEN, USA) supplemented with complete protease and phosphatase inhibitor cocktail (ASPEN, USA). We used Bicinchoninic acid (BCA) assay (ASPEN, USA) to estimated protein concentration after centrifugation at 13000 rpm for 5 min. Proteins were separated on SDS-polyacrylamide gels and transferred to PVDF membranes. Then, the membranes were incubated overnight at 4 °C with the following primary antibodies: rabbit monoclonal anti-GAPDH antibody (diluted 1:10000, Abcam), rabbit monoclonal anti- p-AMPKα1 antibody (diluted 1:1000, Abcam), rabbit monoclonal anti- PGC-1α antibody (diluted 1:500, Abcam), rabbit monoclonal anti-PPARα antibody (diluted 1:1500, Abcam), rabbit monoclonal anti-FAT antibody (diluted 1:500, Bioss), rabbit monoclonal anti-VLCAD antibody (diluted 1:1000, Abcam) and then with secondary HRP-goat anti-rabbit antibody (diluted 1:10000, ASPEN) for 30 min at room temperature (RT). For loading controls, the membranes were stripped with stripping buffer (ASPEN, USA) for 10 min at room temperature. Antibody binding was detected with the ECL detection reagent (ASPEN, USA). At last, we quantified bands with AlphaEaseFC Software and data are shown as the ratio of total protein to GAPDH normalized to control.

### Real-time quantitative PCR

30–50 mg LAA tissues were homogenized in TRIZOL (Invitrogen, Carlsbad, CA) for extraction of RNA according to the manufacturer’s protocol. Both reverse transcription and quantitative PCR were carried out with Promega kits (Promega, Madison, WI). The StepOne Real-Time PCR (Life Technologies, Alameda, CA) was used for real-time qPCR analysis. The primers of gene PPARα, ACC, CPT-1, VLCAD, and GAPDH were synthesized from GeneCreate (GeneCreate, Wuhan, China). The primer sequences used are listed in Table 1. The amount of mRNA and normalized to the amount of GAPDH. The primers designed in RT-PCR were trans-intron, so that the template of amplified products could be distinguished from that of RNA or genomic DNA. When extracting supernatant after centrifugation, make sure not taking the middle membrane and the liquid below. The extracted RNA was digested by DnaseI (1 h, 37 degrees) to ensure that the DNA is removed.

**Table 1:** Sequences of primers used for real-time PCR

Primer	Forward (5′ → 3′)	Reverse (5′ → 3′)
PPARα	TAAAGAGCCTAAGGAAACCGTTC	GCAAATGATAGCAGCCACAAA
CPT-1	AACCCGAACATTCCATACCC	GAACGCACAGTCTCCGTCC
ACC	AAAGGATTCCCGTACAAGCAG	CAGTCCACCCGAAGACCACT
VLCAD	CATAGCTGCTTTCTGTCTAACGG	AGGCTTTGATGCCCATCTTC
GAPDH	GAAGGTCGGAGTGAACGGATT	CATTTGATGTTGGCGGGATC

### Electrophysiological measurements

The electrophysiological parameters, including effective refractory period (ERP) and ERP dispersion (ERPd), were measured as previous studies described [23], programmed stimulation of atrial myocardium was performed by using the computer-based Lab System (Lead 7000, Jingjiang, Chengdu City, China). ERP was determined by using programmed pacing, which consisted of 8 consecutive stimuli (S1-S1 = 300 ms) followed by a premature stimulus (S1-S2), which was progressively decremented until atrial refractoriness was achieved. Pacing was performed at 2 × threshold (TH). ERP d was calculated offline as the coefficient of variation (standard deviation/mean) of the ERP at all recording sites.

### Statistical analysis

Statistical analyses were performed using R-3.4.3 (https://www.r-project.org/) All the values were expressed as mean ± SEM. The statistical significance of differences between the means was assessed by ANOVA and Tukey HSD for comparisons between two groups. A difference at *P* < 0.05 was considered statistically significant.

## Results

### Metformin prevents ARP-induced fatty acid metabolic disorders in LAA

**Fig. 1:**
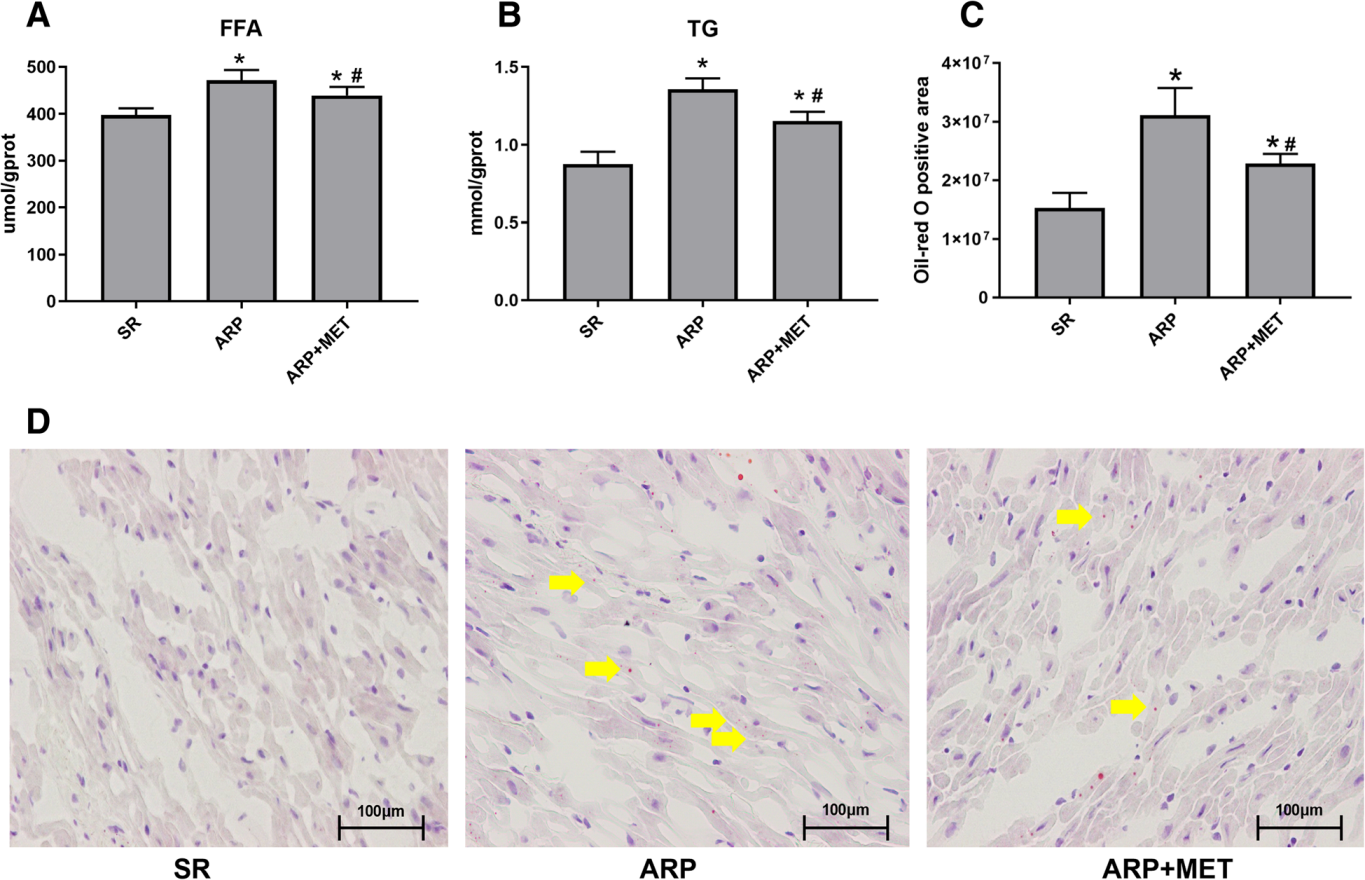
Metformin decreases ARP-induced lipid accumulation in LAA. **a**, **b** Extract LAA lipids as shown in Materials and Methods, and detect the triglyceride and free fatty acid contents. Data are shown as mean ± SEM. **c** Quantitative analysis of oil red O positive area. **d** Representative images of oil red O staining for lipids (red color, × 40). SR, sinus rhythm; ARP, atrial rapid pacing; MET, metformin. *P <0.05 versus SR group; ^#^*P* <0.05 versus ARP group; *n* = 5 per group. (The arrow points to the lipid droplets which stained with red color)

We examined the effect of metformin on FA metabolism. The concentration of FFA and TG in LAA increased markedly (P < 0.05, P < 0.05, respectively) at the ARP group, while Metformin treatment effectively alleviates these effects (Fig. 1a, b). Use Oil Red O staining to analyze the neutral lipid contents in LAA tissues. As shown in Fig. 1d, ARP group’s LAA tissues had tremendous lipid droplets stained with a red color; metformin effectively decreased the lipid accumulation, which was also quantified in Fig. 1c. These data implied that metformin alleviates ARP-induced fatty acid metabolic disorders in LAA.

### Metformin regulates key fatty acid metabolic factors in protein level

**Fig. 2:**
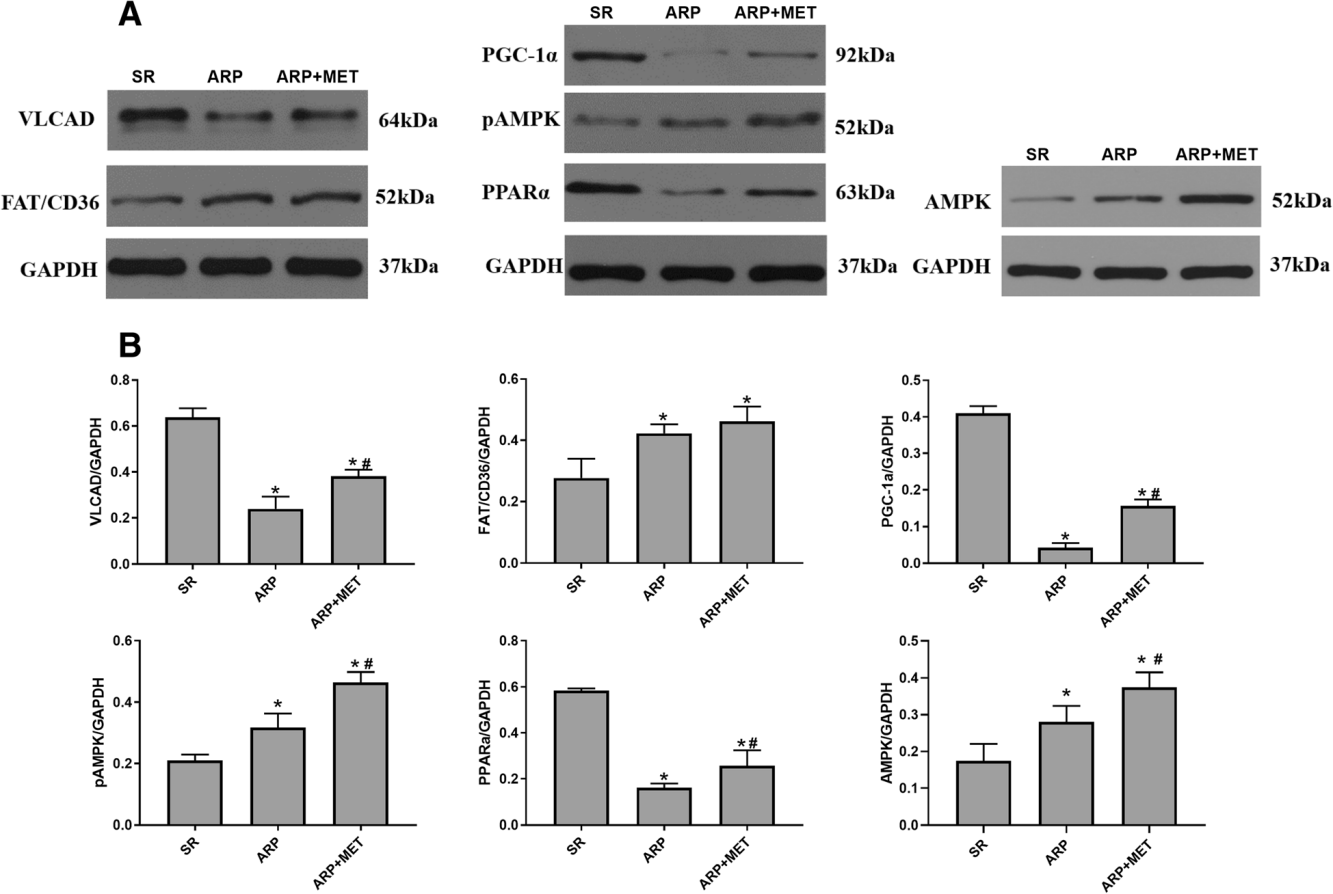
Alterations in the protein expression of key metabolic factors in the left atrial appendage. **a** Representative images of the protein expression of AMPK, pAMPK, FAT/CD36, PPAR-α, PCG-1α, and VLCAD in the left atrial appendage of canines. **b** Quantitative analysis of the expression of AMPK, pAMPK, PPAR-α, PCG-1α, FAT/CD36 and VLCAD proteins in the left atrial appendage. Data are shown as mean ± SEM. SR, sinus rhythm; ARP, atrial rapid pacing; MET, metformin; AMPK, adenosine 5′-monophosphate (AMP)-activated protein kinase; pAMPK, phosphorylatied adenosine 5′-monophosphate (AMP)-activated protein kinase; FAT/CD36, fatty acid translocase; PGC-1α, peroxisome proliferator-activated receptor-gamma coactivator 1α; PPAR-α, peroxisome proliferator-activated receptor; VLCAD, Very long-chain specific acyl-CoA dehydrogenase.*P < 0.05 versus SR group; ^#^*P* < 0.05 versus ARP group; *n* = 5 per group

We examined the protein expression of key metabolic factors in LAA from three groups (Fig. 2a, b). Exposure of the atria in canines to rapid pacing 6 h obviously reduced the expression of VLCAD and increased AMPK, pAMPK and FAT/CD36 expression; The expression of PPAR-α and PGC-1α were significantly down-regulated in the ARP group compared with the SR group, which was attenuated by metformin. The expression of AMPK and pAMPK was up-regulated in ARP group, and significantly up-regulated at MET+ARP group. No significant difference in FAT/CD36 expression level was found between the ARP group and MET+ARP group, but the expression of VLCAD expression significantly increased in MET+ARP group compared to ARP group. These data indicate that ARP causes a mismatch between FA uptake and FA oxidation in LAA, characterized by increased FA uptake but decreased FAO. Treatment with metformin helps enhance the oxidation of FA but doesn’t affect much on its uptake.

### Metformin regulates key fatty acid metabolic factors in transcription level

**Fig. 3:**
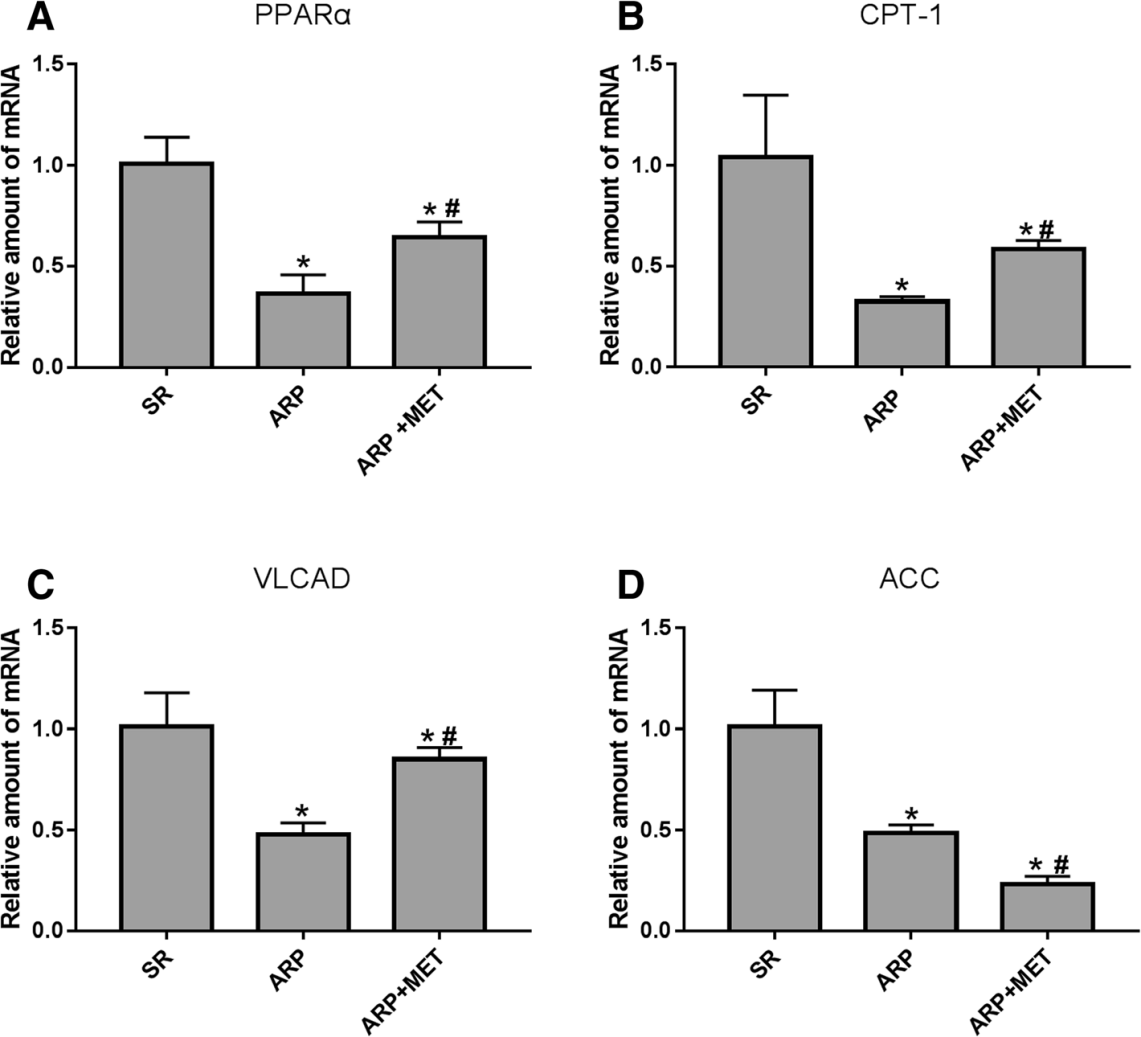
Alterations in the mRNA expression levels of key metabolic factors. Four lipid oxidation gene expression were examined by the RT-qPCR. **a**-**c** shown, PPAR-α, carnitine palmitoyl transferase (CPT)-1 and VLCAD were down-regulated in ARP group, but metformin attenuate those alterations. **d** shown, transcription level of ACC was decreased in ARP group and significantly decreased in MET+ARP group

To investigate the inhibitory effect of metformin on atrial lipid metabolic remodeling and the relationship between PPAR-α and VLCAD, some lipolytic gene expression were examined by the RT-qPCR. As shown in Fig. 3a-c, PPAR-α, carnitine palmitoyl transferase CPT-1, and VLCAD were down-regulated in ARP group, but metformin attenuated those alterations. As shown in Fig. 3d, the transcription level of ACC was decreased in ARP group and significantly decreased in MET+ARP group, which was negatively related to pAMPK protein expression (Fig. 2a). Both studies of AF indicated a significant down-regulation of transcripts and proteins involved in lipid metabolism [24]. Moreover, recent studies indicated that AF induced an increase of factors involved in lipid droplet formation [25]. Liu et al. [26] observed that the levels of mCPT-1 and MCAD were markedly down-regulated in animal model and humans with AF. At the same time, the results also indicated that AF induced the accumulation of lipid droplets by downregulating fatty acid oxidation.

### MET could improve the electrophysiological disorders caused by atrial-tachypacing

**Fig. 4:**
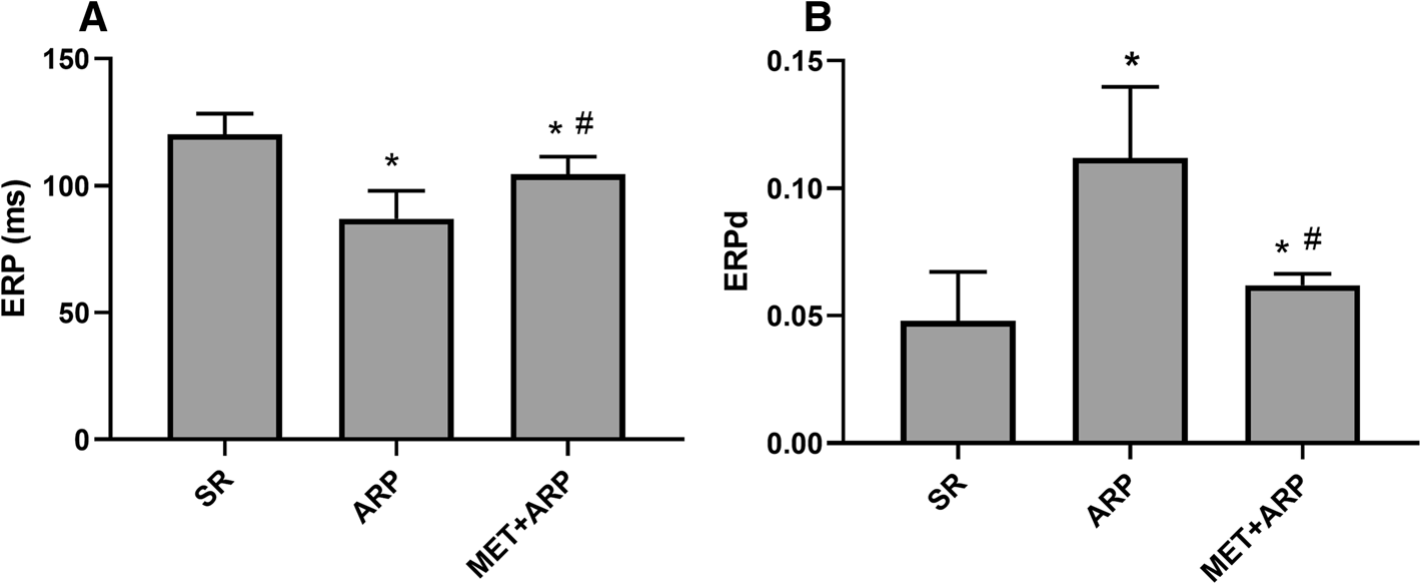
Alteration of electrophysiological parameters after ARP and the effect of MET. Alteration of AERP (A) and AERPd (B) after 6-h ARP or MET treatment. *P <0.05 vs. SR group; ^#^*P* <0.05 vs. ARP group. ARP, atrial rapid pace; MET, metformin

The atrial effective refractory periods (ERPs) at the LAA and LA are shown in Fig. 4a. Compared with the SR group (120.20 ± 8.15 ms), the ERPs at all recording sites was significantly decreased after 6 h rapid atrial pacing (86.70 ± 11.06 ms), whereas treatment with MET (104.50 ± 7.04 ms) attenuated the decrease in ERPs (*P* < 0.05 for all). On the other hand, dispersion of ERP in ARP group was significantly higher than sham-operated group (0.11 ± 0.03 vs 0.04 ± 0.02, *P* < 0.05), whereas MET treatment reversed the dispersion increase (0.06 ± 0.01 vs. 0.11 ± 0.03, *P* < 0.05) (Fig. 4b).

## Discussion

The main findings of our present study were that: Firstly, the PPAR-α, PGC-1α, VLCAD expression levels were decreased, while the pAMPK and FAT/CD36 were increased in the canine model of AF; Secondly, metformin increased pAMPK expression in canine model of AF, and prevented the down-regulation of PPAR-α, PGC-1α, VLCAD; Third, metformin prevented the atrial metabolic remolding of AF partly through the AMPK/PPAR-α/VLCADS pathway.

FA oxidation (FAO) supplies 60 to 80% of myocardial ATP in the healthy adult mammalian heart, whereas the balance (20 to 40%) comes from glucose oxidation. 2% or less is derived from the catabolism of lactic acid and ketone bodies [27]. The concept of myocardial lipid metabolic remodeling was first raised by van Bilsen M [28] including the alteration of HEPs (High-energy phosphates) [3], mitochondrial function and metabolic substrates. HEPs include adenine nucleotides and creatine phosphate. Recent years, many proteomics and metabolomics studies have found that disordered energy metabolism existed in the myocardium of patients with AF, in other words, lipid metabolic remodeling might also be involved in the development of AF [5, 29, 30]. It showed that abnormal lipid metabolism plays an important role in the occurrence and development of atrial fibrillation.

Circulating FAs enter into cardiomyocytes via the FA transporter FAT/CD36 and then entry into mitochondria for FA oxidation through Carnitine palmitoyl transferase-1 (CPT-1). The mitochondrial fatty acid β-oxidation (FAO) pathway plays an important role in ATP production in many high-energy demand tissues [31]. In diabetic myocardium, the capacity of fatty acid uptake far exceeds the heart to consume, promoting triacylglycerol and ceramide deposition, eventually leading to myocardial steatosis and cardiac hypertrophy [32], which indicates that imbalanced fatty acids metabolism is related to lipid deposition caused by AF. Our previous proteinomics study showed that some proteins involved in lipid metabolism were downregulated including dehydrogenase catalyzes [5], and one of them is VLCAD, the initial rate-limiting enzyme in mitochondrial fatty acid β-oxidation, which may play a key role in the regulation of lipid metabolism. This result is consistent with our study: compared with SR group, pAMPK and fatty acid translocase (FAT/CD36) increased, but the levels of the rate-limiting enzyme VLCAD expression decreased significantly. In ARP group, the increase of lipid uptake but decrease of FAO lead to a mismatch, leading to lipid deposition. What’s more, the accumulation of triglyceride, and subsequent increased toxic intermediates production, contribute to decreased adenosine triphosphate (ATP) synthesis, increased formation of reactive oxygen species (ROS), mitochondrial uncoupling, and finally apoptosis [33]. Abnormal FA metabolism may disturb atrial conduction and benefit the development and persistence of re-entry circuits [34]. This also supports the decrease of high-energy phosphoric compounds and the change of electrophysiological parameters in ARP group, suggesting that the disordered of mitochondrial FAO is closely related to the occurrence and development of atrial fibrillation.

AMPK regulated cardiac energy homeostasis, and is regarded as a sensor of metabolic stress. It has been wildly noted the AMPK has a protective effect on the development of critical pathologies like myocardial ischemia, cardiac hypertrophy, diabetic cardiomyopathy, and heart failure [35]. Recent research suggests a protective effect of AMPK on atria structure remodeling [14]. Current research found that MET, AICAR (5-aminoimidazole 1 carboxamide ribonucleoside) can activate AMPK activity [36], while complex C inhibits AMPK activity [27]. Metformin exerts cardiovascular protection through AMPK dependent and independent pathway. Recent studies have suggested that metformin could inhibit cardiomyocyte apoptosis induced by H2O2 [22] and alleviate ischemia-reperfusion injury through AMPK activation [37]. Fu et al. [38] demonstrated that metformin attenuates cardiac hypertrophy induced by pressure overload in nondiabetic mice and the effect may dependent on AMPK activation. Futher more, metformin has a direct effect on suppressing proinflammatory responses in an AMPK-independent manner [39]. MET can also inhibit myocardial cell lysis and oxidative stress, and reduce peripheral blood TG and TC in pacing HL-1 cell [18]. All of these pathways may be the basis of the phenomena observed in our study. Studies have confirmed that oral administration of MET 2 weeks (100 mg/k/ day) can activate AMPK activity in the heart [22–41]. A recent article shows that phosphorylated AMPK is decreased in chronic AF, while it is increased in paroxysmal AF [14]. It indicates that the initial increase of pAMPK may be an adaptive/compensatory response to energy deficiency during the onset of acute AF. While in the chronic AF, the slightly increased pAMPK can no longer meet the accumulating high energy demand so that the level of pAMPK begin to decrease and reverse to a decompensatory phase, and it is the continuous decrease of pAMPK that promote the development of AF. The mechanism of AMPK activation adaption to AF is still not clear. In our study, compared with the SR group, pAMPK increased slightly in ARP group but significantly increased in MET + ARP group. Therefore, it can be concluded that by using metformin in the acute phase to further increase the activation of pAMPK, the energy deficiency and electrical remodeling is reversed, thus inhibiting the progression of atrial fibrillation..

The genes involved in heart energy metabolic pathway are transcriptionally regulated by members of the nuclear receptor superfamily, specifically the peroxisome proliferator-activated receptors (PPARs) and the nuclear receptor coactivator, PPARα coactivator-1 (PGC-1α) [42, 43]. PPAR-α is an important regulator of fatty acid metabolism. Their target genes participate in lipid metabolism. Activation of PPAR-α induces uptake, utilization, and catabolism of fatty acids through the upregulation of gene expression involved in fatty acid transport, binding, and activation, and increases mitochondrial fatty acid β-oxidation [44–47]. VLCAD, the key rate-limiting step in the initial of mitochondrial fatty acid β-oxidation, is also regulated by PPAR-α. PPAR-α activation has been proved to up-regulate fatty acid metabolism genes and reverse cardiac dysfunction [48, 49]. Compared with ARP group, the expression of PPARα, PGC-1α and VLCAD in MET+ARP group increased, while the expression of FAT/CD36 has no significant change. Together with decreased LAA lipid deposition and the recovery of high-energy phosphoric compounds and ERP our result suggest that metformin facilitate the beta-oxidation of fatty acids through the AMPK pathway, and improves energy supply and electrical conduction abnormalities. This is consistent with the reports in previous studies as metformin promotes the expression and activation of PGC-1α to improve mitochondrial function [50]. And AMPK-mediated increased activation of PGC-1a lead to improved metabolism of fatty acids [51] and more-efficient energy utilization [50].

We suspected that the cardioprotective impact of metformin in AF might be mediated by the change of the gene expression in cardiomyocytes to a state that favorslipid oxidation, as Fig. 4 shown. During the process of AF, the fatty acids metabolism in LAA is altered, mainly characterized by the abnormal expression of the rate-limiting enzyme; The FA uptake in cardiomyocytes is increased but β-oxidation of fatty acids in the mitochondria is weakened, leading to a mismatch. Metformin regulates cardiac energy homeostasis by promoting the β-oxidation of FA in AF partially through AMPK/PPAR-α/VLCAD pathway and reducing lipid accumulation. The present study may provide a novel therapeutic strategy for AF.

## Conclusions

We verify that rapidly pacing induced profound changes of lipid metabolism with characteristics similar to alterations observed in the human atria, mainly characterized by the abnormal expression of lipids metabolism rate-limiting enzyme, and abnormal concentration of lipid metabolic substrates. and demonstrated that the AMPK/PPAR-α/ VLCAD pathway participates in atrial fatty acid metabolic regulate during AF. Our study was conducted under a non-diabetic condition in an animal model. Using metformin to activate AMPK and PPARα in rapid paced canine model which break the vicious cycle of “AF begets AF” in the beginning stage of AF to prevent these effects. All of the data suggest that MET may be benefit to the lipid metabolic remodeling of AF. and may become a novel target in AF therapy which deserves further research.

## Limitations

There were a few limitations in this study, we only recorded the changes in energy metabolism by using AMPK activator. Whether the inhibitor of AMPK or PPARα blocked those benefits was not investigated. Further studies are needed to verify the effect of MET in myocardium cells level.

## Abbreviations

AF: Atrial fibrillation
AMPK: AMP-activated protein kinase
ARP: Acute Rapid Pacing
ATP: Adenosine triphosphate
ERP: Effective refractory period
ERPd: Effective refractory period dispersion
FA: Fatty acid
FAO: Fatty acid oxidation
LAA: Left atrial appendage
MET: Metformin
p-AMPK: Phosphorylated AMP-activated protein kinase
PGC-1α: Peroxisome proliferator-activated receptor-α coactivator-1
PPAR-α: Peroxisome proliferator-activated receptor-α
VLCAD: Very-long chain acyl-CoA dehydrogenase
